# Face recognition improvements in adults and children with face recognition difficulties

**DOI:** 10.1093/braincomms/fcac068

**Published:** 2022-03-22

**Authors:** Sarah Bate, Kirsten Dalrymple, Rachel J. Bennetts

**Affiliations:** 1 Department of Psychology, Faculty of Science and Technology, Bournemouth University, Poole BH12 5BB, UK; 2 Institute of Child Development, University of Minnesota, Minneapolis, MN 55455, USA; 3 Division of Psychology, Department of Life Sciences, Brunel University, London UB8 3PH, UK

**Keywords:** face recognition, face perception, training, remediation, prosopagnosia

## Abstract

While there have been decades of clinical and theoretical interest in developmental and acquired face recognition difficulties, very little work has examined their remediation. Here, we report two studies that examined the efficacy of an existing face training programme in improving face-processing skills in adults and children with developmental face recognition impairments. The programme has only been trialled in typical children to date, where 2 weeks of perceptual training (modelled on an adapted version of the popular family game *Guess Who?*) resulted in face-specific improvements for memory but not perception after 2 weeks of training. In Study 1, we performed a randomized, parallel groups, placebo-controlled trial of the same programme in 20 adults with a pre-existing diagnosis of developmental prosopagnosia. Assessment tasks were administered immediately before and after training, and 2 weeks later. Face-specific gains in memory (but not perception) were observed in the experimental group and were greatest in those with the poorest face recognition skills at entry. These gains persisted 2 weeks after training ceased. In Study 2, a case-series approach was used to administer the experimental version of the training programme to four children who presented with difficulties in face recognition. Improvements in face memory were observed in three of the participants; while one also improved at face perception, there was mixed evidence for the face specificity of these gains. Together, these findings suggest plasticity in the human face recognition system through to at least mid-adulthood and also pave the way for longer-term implementations of the face training programme that will likely elicit greater gains in both adults and children.

## Teaser

Following a face training programme, Bate *et al.* reported an improvement in face memory in 10 adults with developmental prosopagnosia. Four children with face recognition difficulties also completed the programme, and three improved at face memory. These findings indicate plasticity in the face recognition system at least through to mid-adulthood.

## Graphical Abstract

**Graphical Abstract:**
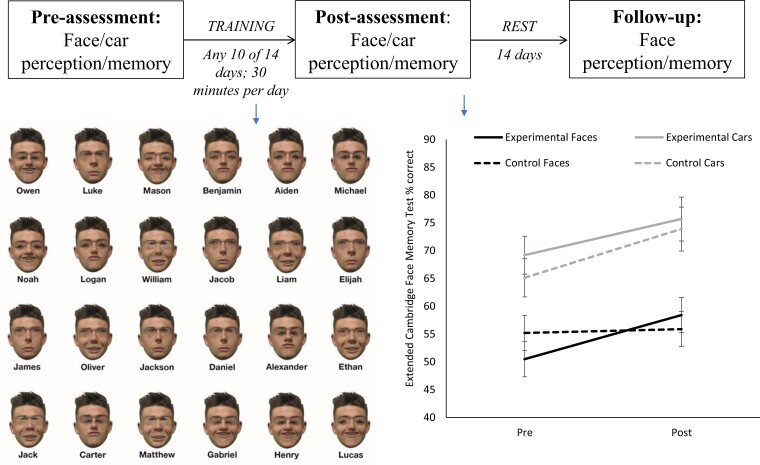

## Introduction

Difficulties with face recognition, a condition known as prosopagnosia or face blindness, have long been reported in adults and children.^1,2^ While it is relatively rare to acquire prosopagnosia following neurological insult,^3^ many more people experience a developmental form of the condition^4,5^ where face recognition difficulties are apparent at least from early childhood.^6^ It is well accepted that developmental prosopagnosia can be heterogeneous in presentation^6^; while the hallmark symptom of the condition is a difficulty in remembering faces, there is more variability in the ability to perceive faces.^7^ That is, while some individuals with prosopagnosia can accurately judge facial characteristics such as gender, age or emotional expression, others cannot. A substantial amount of research has investigated whether other perceptual processes are also impaired in developmental prosopagnosia, such as the processing of spatial relationships within faces (configural processing) or identification of specific facial features (featural processing).^8^ Although the results are somewhat heterogeneous, there is some evidence to suggest that both processes can be impaired; for example, both sensitivity to spacing changes^9,10^ and the effects of rotating a face 180° (inversion, which is thought to disrupt holistic and configural processing)^8^ are less pronounced in typical perceivers than in individuals with developmental prosopagnosia.^11,12^ Further, some individuals have difficulty recognizing inverted faces (which, in theory, rely on featural processing)^8^ or identifying subtle alterations to facial features.^9,10,12^

Despite widespread theoretical and clinical interest in prosopagnosia across the lifespan,^1,13^ little work has attempted to improve face recognition difficulties.^14,15^ While some studies have developed compensatory strategies that aim to circumvent failures in identity recognition,^16–18^ few studies have attempted to remediate underpinning processing strategies. Yet, modest gains have been observed in existing remedial programmes. DeGutis *et al.*^19^ trained 24 adults with developmental prosopagnosia via a 3-week online programme that targeted relational processing^20^—the ability to process spatial relationships between different facial features. Participants were required to make rapid category judgements about large numbers of faces for 30–40 min per day, by integrating the distance between the eyes and eyebrows with the distance between the mouth and nose. Compared with performance in a no-training waiting condition, participants showed moderate improvements on measures of front-view face discrimination, tests of holistic processing and in self-reported diaries of everyday face recognition experiences. The largest improvements were observed in those who dedicated more time to training.

Davies-Thompson *et al*.^21^ applied a similar training programme to 10 adults with acquired prosopagnosia over an 11-week period. These participants were required to discriminate whole-face differences over a variety of views and expressions for 30–40 min per session, three times a week, and a staircase design controlled the difficulty levels of subsequent trials. Gains generalized to new viewpoints and expressions of the trained faces and to untrained faces, and persisted for at least 3 months post-training. While there were minimal gains on standard tests of face processing and in transfer to everyday life, gains were greater for individuals who had initially presented with more severe deficits. Corrow *et al*.^22^ administered the same training programme to 10 adults with developmental prosopagnosia. Again, the improvements generalized to untrained expressions and views of the target faces, with some evidence of transfer to untrained faces, standardized face recognition tasks and face recognition skills in everyday life.

The studies reviewed above suggest that adult cases of developmental prosopagnosia can benefit to some degree from training, with both the severity of face-processing deficits at entry and level of engagement influencing training outcome. There is comparatively less evidence of the effects of face-related training in children. Tanaka *et al*.^23^ reported small face-processing training gains in children with autism spectrum conditions following 20 h of a computer-based programme. However, these were limited to a small subset of tasks and did not generalize to tasks assessing matching across expressions and orientation, or face memory. To date, the only existing remedial training study in a child with prosopagnosia was reported in the case of EM, a 14-year-old female who acquired face recognition difficulties following encephalitis at the age of 8 years.^24^ EM underwent 14 weeks of perceptual training via an online face perception programme that attempted to improve her ability to make fine-grained discriminations between faces, progressing across 10 levels of difficulty. EM’s face perception skills improved post-training, and she spent more time viewing the inner facial features. The gains transferred to new faces, and laboratory assessments also indicated improvements in her recognition of personally known faces, although this did not transfer to daily life.

**Figure 1:**
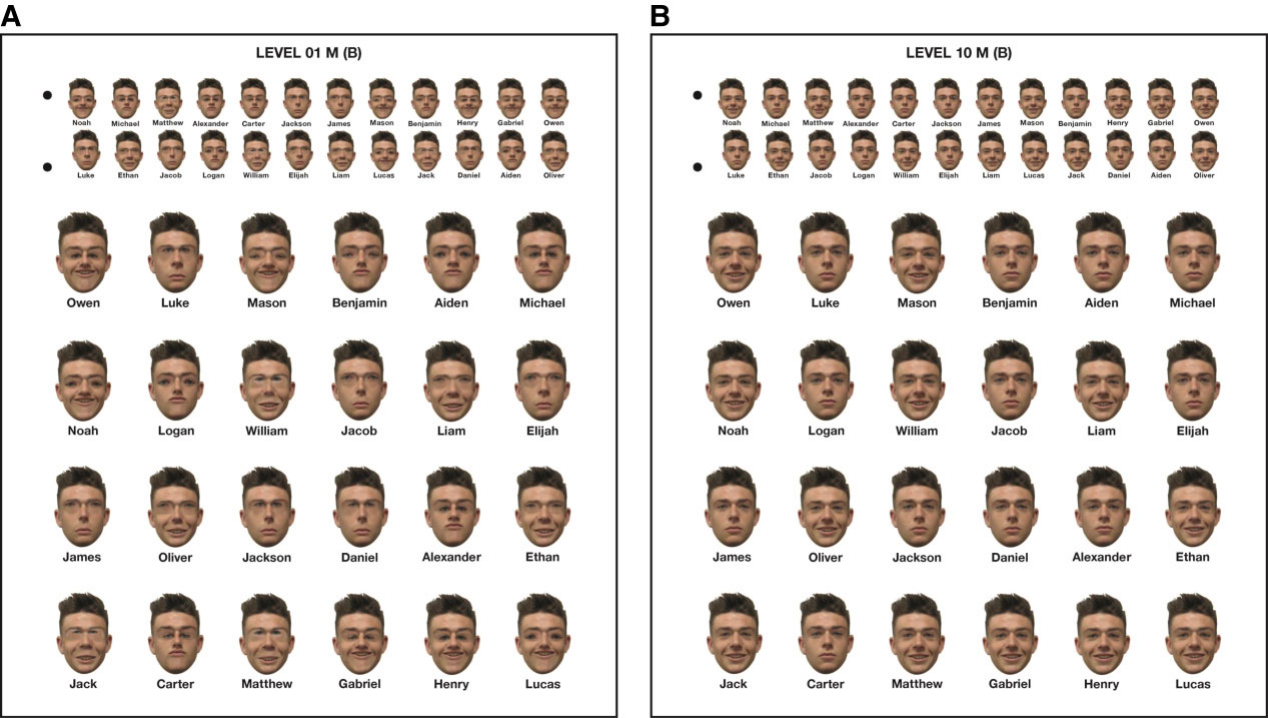
**Sample training stimuli from the experimental condition.** The images show male stimuli cards for (**A**) training Level 1 and (**B**) training Level 10.

More recently, we created a new face training programme that built upon the combined principles of the existing remediation programmes described above and was framed within a commercial family game (*Guess Who:* Hasbro Gaming) to encourage engagement.^25^ In a randomized placebo-controlled design, 81 typical children aged 4–11 years took part in the new experimental training programme or an active control condition. Over 10 training sessions, experimental participants were required to discriminate between faces that differed in feature size or spacing across 10 levels of difficulty (see Fig. 1), whereas control participants continuously played the standard version of *Guess Who* within the same timeframe. Improvements in face memory but not face matching were observed in the experimental compared with the control group, and there were no gains on tests of object matching or memory. Face memory gains were maintained at a 1-month follow-up, consistent across age and larger for poorer perceivers.

Having provided evidence for the effectiveness of the *Guess Who* training programme in typical children, the current study aimed to examine whether it could remedy face recognition difficulties in both adults and children. Given (i) the much wider availability of adults with developmental prosopagnosia and (ii) the wider availability of reliable tasks of face recognition ability for adults, Study 1 implemented a placebo-controlled intervention study using adults previously confirmed to have this condition. Because there are few children with face recognition difficulties available for research participation, a second study used a case-series approach to examine the efficacy of the experimental version of the training programme in four children with face recognition impairments.

Given the benefits in typically developing children were found to be limited to face memory, we continued to assess memory and perception in both studies to examine the replicability of this finding. The proposed independence of face perception and face memory has long been investigated in the adult neuropsychological literature and is reflected in dominant theories of face processing,^26,27^ as well as differential neuropsychological profiles of both acquired and developmental prosopagnosia.^7,28,29^ It is therefore pertinent to examine whether gains from the programme extend to face perception in some or all individuals with face recognition difficulties. Finally, we examined whether the gains from the programme are face-specific or extend to other categories of the object. The issue of domain-specificity has been debated in the adult cognitive neuropsychological literature for more than 50 years but is far from resolved.^30^ Some research on acquired and developmental prosopagnosia suggests that face and object processing deficits can sometimes dissociate,^31–33^ and it is of interest here to examine whether face training in impaired participants acts on face-specific (as in typical children)^25^ or more general mechanisms.

## Study 1

An initial study replicated the randomized placebo-controlled design used with typical children in our existing work,^25^ applying it to adults with developmental prosopagnosia using reliable, dominant assessment tasks of the face and car memory and perception.

### Materials and methods

#### Participants

Based on the sample size used in the previous work,^19,21,22^ we set out to recruit at least 20 adults with a pre-existing diagnosis of developmental prosopagnosia^34^ (for inclusion data see Supplementary Table 1). A total of 22 participants volunteered to take part in the study in exchange for a small financial incentive. Using a parallel-group design with an allocation ratio of 1:1, the lead author randomly allocated participants to either the experimental or control condition (a random-number generator was used to create a list of places across the two conditions, and participants were assigned to the next available slot at recruitment). Two participants dropped out during the central training phase, both from the control condition. Recruitment paused when 10 participants in each training condition had successfully completed the study through to the post-assessment phase, resulting in 10 participants in each condition (experimental condition: five females, *M* age = 41.2 years, SD = 11.9; control condition: nine females, *M* age = 45.6 years, SD = 11.1). There was no difference in age between the two conditions, *t*(18) = 0.856, *P* = 0.403, nor in their Cambridge Face Memory Test (CFMT) or Cambridge Face Perception Test (CFPT) performance at screening, *t*(18) = 1.354, *P* = 0.096, and *t*(18) = 0.962, *P* = 0.175, respectively. Ethical approval was granted by the institutional Ethics Committee, and all participants provided their informed consent according to the Declaration of Helsinki.

### Materials

#### Training

The training procedure replicates that reported in detail in Bate *et al*.,^25^ adopting the format of the popular two-player family game *Guess Who*. In brief, two players sit opposite each other, viewing a plastic frame composed of 24 closable plastic windows, each displaying a face and a name. Faces are displayed in colour from the neck upwards, measuring ∼2.0 × 1.8 cm. At the start of the game, all windows are open, and each player selects one face. They then take turns to ask yes/no questions that will allow them to progressively close the windows of faces that are inconsistent with their opponent’s chosen face until only one remains. The standard version of the game uses animated faces that differ according to sex, ethnicity, hair or eye colour and the presence of spectacles or hats. This version was used as an active control condition for the training programme, as in our previous work.^25^

For the experimental training condition, we developed new insert cards that slot into the same plastic frame. Two new versions were created: one displaying male faces and the other displaying female faces. Each version was created from two colour photographic images of a model, one displaying a neutral facial expression and the other a happy expression. Twelve manipulated images were then created from each base image by varying combinations of four adjustments: two that manipulated the spacing of facial features (the distance between the eyes, or the distance between the eyes and mouth) and two that manipulated the size of specific facial features (the eyes and/or nose). This resulted in 24 new targets for each of the male and female insert cards, and each was paired with a name taken from the annual UK list of popular baby names.

The images displayed in each version were then adjusted to produce nine further image cards, where the manipulations became progressively less extreme (i.e. expressions became more ambiguous, or the differences in spacing/feature size became smaller). This resulted in 20 different image cards (10 males, 10 females; with 10 levels of difficulties for each sex). Two versions of the cards were created, where the location of each target was randomly dispersed to avoid location-based cueing. Because the external features of each target were identical, players could only discriminate their opponent’s target face by referring to the expression or feature spacing/size manipulations described above (see Fig. 1). For instance, they might ask if the face is happy, if the eyes are large, or if the eyes are close together.

#### Assessment tasks

In addition to the training task itself, four existing tasks with high reliability^5,35,36^ (see Bate *et al*.^37^ for previous use in a training study) were used to assess the efficacy of training, assessing face and object (car) processing for memory and perception (the extended version of the CFMT^38^; the CFPT^39^; the Cambridge Car Memory Test^40^ and the Cambridge Car Perception Test)^41^. These are dominant tasks that are frequently used in the face-processing literature and are all well detailed elsewhere.

While all participants had previously completed the short version of the CFMT and the upright version of the CFPT, we opted to reuse these tasks due to (i) their excellent psychometric properties, including good test–retest reliability with only small practice effects in typical perceivers,^36^ (ii) the fact that it had been at least a year since participants had previously completed the tasks, (iii) the longer format of the CFMT+ offers 30 new harder trials that our participants would not have previously been exposed to and (iv) the inverted trials of the CFPT also allowed an inversion effect to be calculated. In typical perceivers, inverted faces are thought to be processed in a different manner to upright faces. Specifically, inversion disrupts holistic processing (the integration of information from across the face, considered a hallmark of typical face perception)^8^ and configural processing (sensitivity to spatial relationships between features),^8,42^ resulting in a more piecemeal, feature-based strategy^43^ and poorer performance compared with upright faces (the face inversion effect). This inversion effect is often reduced in individuals with developmental prosopagnosia,^12,40^ suggesting some impairment in holistic or configural processing. Therefore, the inversion effect acted as an indirect measure of whether training influenced the processing mechanisms underpinning face recognition.

Further, as sufficient multiple versions of the tests are not currently available, we re-administered the identical tasks at each assessment stage of the study (see below). While test–retest effects were expected, the use of a control training condition, and object as well as face tasks, allowed us to examine whether performance on the face tests improved in the experimental group over and above the control group, and in faces over and above objects. This also follows the protocols we previously used with typical children, facilitating comparison with those findings.^25^

#### Procedure

All participants initially completed the four assessment tasks (face and car memory and perception) online, in their own homes. The two memory tasks were always completed first, with the order of the face and car tests counterbalanced for both memory and perception. Participants then immediately entered a 14-day training period, in which they were required to play the relevant version of *Guess Who* for at least half an hour per day, on any 10 of the 14 days. Opponents were other adults without face recognition difficulties from their own household, and training occurred within their own home.

Participants in the control condition simply played the commercial version of the game, repeatedly using the same animated stimuli cards throughout. Those in the experimental condition began with the male version of the new Level 1 stimuli cards, otherwise following the game’s standard protocols. The participant was deemed to have correctly performed the task whenever a successful guess was made by either of the two players (the experimental participant would need to answer the questions of their opponent correctly in order for the opponent to win). When the game was successfully completed on two consecutive occasions, the players switched their cards to the Level 1 female version. When that level had been won on two consecutive occasions, they proceeded to Level 2, and so on, alternating between the male and female versions at each level.

**Figure 2:**
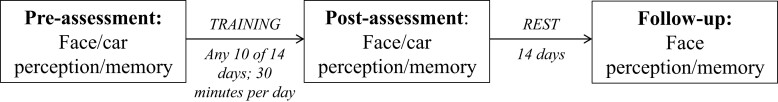
**The training procedure for all participants.** The timeline shows the spacing of assessment sessions relative to the training period, which was identical in both the experimental and control condition.

Following the 14-day training period, participants in both conditions immediately completed the four face and car memory and perception assessment tasks a second time (the same protocols were used for order and counterbalancing as in the pre-assessment). Both experimental and control participants were then asked to refrain from playing any version of *Guess Who* for the next 2 weeks, after which they completed the face (but not the car) memory and perception tasks a final time (memory test first), to see if any face-processing gains were maintained. While this differed from our original study using typical children (where a 4- rather than 2-week rest period was implemented between post-assessment and follow-up),^25^ we chose to reduce this period due to uncertainty regarding the length of the COVID-19 lockdown at the time of testing, in order to avoid any potential confounds that may affect the data via changes in social contact. The entire procedure is summarized in Fig. 2.

#### Statistical analyses

Scores on the two memory tests were converted to percentage correct. Performance on the upright and inverted sections of the two perception tasks was calculated independently and converted to percentage correct using the formula {100 × [1 − (total deviation score/maximum score)]}.^33^ An inversion effect was also calculated for the two versions by subtracting the overall accuracy score in the inverted condition from that in the upright condition. The inversion effect was also examined using an inversion index which corrects for differences in baseline performance: (upright − inverted)/(upright + inverted). The pattern of results was the same as simple subtraction; for simplicity, we report subtraction throughout the results. Because this study was conducted during the long-term UK lockdown in the COVID-19 pandemic (March 2020–March 2021), participants were unable to comment on the transfer of gains to their everyday experiences with faces. We therefore only report objective data.

#### Data availability

Data are available from the corresponding author on request.

### Results

#### Engagement and progression

All participants in both conditions completed the training as instructed (i.e. 30 min per day, on any 10 days within the 14-day period). Participants in the experimental condition progressed to at least Level 7 within this time period, with three participants reaching the top level (*M* level reached = 8.50, SD = 1.27). Initial checks on the data confirmed that there were no differences in performance at entry (i.e. the pre-training assessment) between the two training conditions on either the CFMT+ or CFPT upright trials, *t*(18) = 1.048, *P* = 0.154, and *t*(18) = 0.517, *P* = 0.306, respectively.

#### Memory

**Figure 3:**
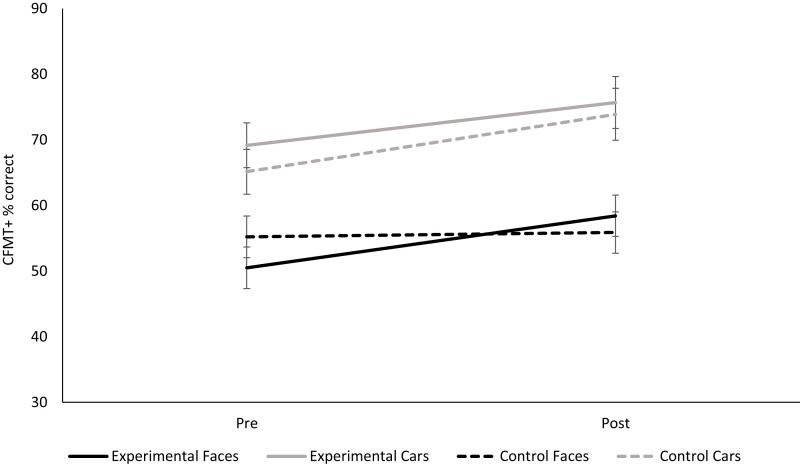
**The gain in the face but not car memory.** The 10 participants in the experimental condition showed an improvement in face memory relative to the 10 participants in the control condition, whereas the improvement in car memory was similar in both conditions. Repeated samples *t-*tests revealed a greater gain in face (but not car) memory performance for the experimental compared with the control condition: *t*(18) = 2.462, *P* = 0.024, *d* = 1.10.

A 2 (condition: experimental, control) × 2 (stimulus: faces, cars) × 2 (assessment: pre-training, post-training) ANOVA, with repeated measurements on the ‘assessment' and ‘stimulus' factors, was carried out on memory scores. While we did not predict a difference in training gains according to the section of the CFMT+ (i.e. noise versus non-noise trials), nor have the power in the statistical model to carry out the relevant ANOVA by section, we recognize that some readers may be interested in this data and report descriptive statistics in Table 1, alongside the results of independent samples *t*-tests comparing the training gain in the experimental versus control conditions. The predicted three-way interaction between condition, stimulus and assessment emerged, *F*(1,18) = 4.849, *P* = 0.041, *ηρ*2 = 0.212 (see Fig. 3). This was followed up with two 2 (condition) × 2 (time) ANOVAs, each considering the face or car memory data.

**Table 1:** Gains in CFMT+ performance following training, according to the test section

Condition	Mean gain (SD)		
	Introduction	Test	Noise
Experimental	62.50	75.00	95.83
Control	35.42	41.67	77.08
*t* ^a^	0.548	1.687	2.844
*P*	0.295	0.054	0.005

^a^ Results of independent samples *t*-tests (d.f. = 18) comparing the gain in each section of the CFMT+ by training condition.

For face memory, there was a significant interaction between time and condition, *F*(1,18) = 6.062, *P* = 0.024, *ηρ*2 = 0.252, superseding a main effect of time but not condition: *F*(1,18) = 8.573, *P* = 0.009, *ηρ*2 = 0.323 and *F*(1,18) = 0.065, *P* = 0.801 (see Fig. 3). A follow-up *t*-test confirmed that the gain in face memory performance was greater in the experimental (*M* = 7.95%, SE = 2.17) compared with the control (*M* = 0.69%, SE = 1.99) group: *t*(18) = 2.462, *P* = 0.024, *d* = 1.10. Further, we carried out an ANCOVA on post-training CFMT+ scores, with pre-training CFMT+ scores entered as a covariate. The ANCOVA confirmed that, after controlling for pre-training performance, the experimental group performed better than the control group in post-training tests of face memory, *F*(1,17) = 4.46, *P* = 0.050 and *ηρ*2 = 0.208. For car memory, a 2 (condition) × 2 (time) ANOVA revealed a significant main effect of time, *F*(1,18) = 14.066, *P* = 0.001, *ηρ*2 = 0.439, but no main effect of condition nor interaction between the two: *F*(1,18) = 0.367, *P* = 0.552 and *F*(1,18) = 0.298, *P* = 0.592, respectively.

The initial, overall ANOVA also revealed a main effect of time that indicated higher accuracy scores for post- (*M* = 65.97%, SE = 1.75) compared with pre- (*M* = 60.00%, SE = 1.55) assessments: *F*(1,18) = 17.841, *P* = 0.001, *ηρ*2 = 0.498. There was also a main effect of stimulus, whereby cars (*M* = 70.97%, SE = 2.41) were recognized better than faces (*M* = 55.00%, SE = 2.11) in all participants, *F*(1,18) = 22.064, *P* = 0.001, *ηρ*2 = 0.551. All other interactions and main effects were not significant (*P*s > 0.140).

**Figure 4:**
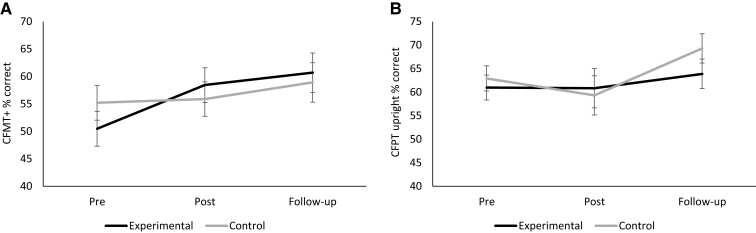
**Face memory and perception scores during the study period.** For the 10 participants in each of the experimental and control conditions, (**A**) face memory and (**B**) face perception scores are shown prior to, immediately after, and 14 days after the training period. Repeated samples *t*-tests on the difference between post-training and follow-up scores for face memory indicated no difference between the experimental and control groups, suggesting the benefit from training was maintained: *t*(18) = 0.286, *P* = 0.778. No-training gains were observed at any time point for face perception (all *P*s > 0.05).

To assess whether gains in face memory were maintained over time, data from the three time points were entered into a 3 (time: pre-assessment, post-assessment, follow-up) × 2 (condition: experimental, control) ANOVA. The interaction approached significance, *F*(2,36) = 3.190, *P* = 0.053, *ηρ*2 = 0.151, and the main effect of time was significant, *F*(2,36) = 9.936, *P* = 0.001, *ηρ*2 = 0.356. The main effect of condition was non-significant, *F*(1,18) = 0.001, *P* = 0.976. Planned linear and quadratic contrasts for the interaction effect were non-significant: *F*(1,18) = 3.072, *P* = 0.097 and *F*(1,18) = 3.446, *P* = 0.080, respectively. A *t*-test on the difference between post-training and follow-up scores indicated no difference between the experimental and control groups: *t*(18) = 0.286, *P* = 0.778. That is, the slight further improvement in face accuracy scores in both conditions likely results from test–retest effects, and no drop-off in gains was observed in the experimental group (see Fig. 4).

#### Perception

A 2 (condition: experimental, control) × 2 (stimulus: faces, cars) × 2 (orientation: upright, inverted) × 2 (assessment: pre-training, post-training) ANOVA, with repeated measurements on the ‘stimulus', ‘orientation’ and ‘assessment’ factors, was carried out on perception scores. The expected stimulus by orientation interaction emerged, *F*(1,18) = 28.697, *P* = 0.001, *ηρ*2 = 0.615. Follow-up comparisons confirmed better performance in the upright (*M* = 61.01%; SE = 2.01) versus inverted (*M* = 48.68%; SE = 1.84) condition for faces but not cars (upright *M* = 63.69%, SE = 1.07; inverted *M* = 63.81%, SE = 1.17): *t*(39) = 6.487, *P* = 0.001, *d* = 1.03 and *t*(39) = 0.117, *P* = 0.907, respectively. This interaction superseded main effects of stimulus (where faces were perceived more poorly than cars; faces: *M* = 54.84%, SE = 1.59; cars: *M* = 63.75%, SE = 1.03) and orientation (where upright stimuli were perceived more accurately than inverted stimuli; upright: *M* = 62.35%, SE = 1.17; inverted: *M* = 56.25%, SE = 1.18): *F*(1,18) = 26.128, *P* = 0.001, *ηρ*2 = 0.592 and *F*(1,18) = 26.988, *P* = 0.001, *ηρ*2 = 0.600, respectively. All other main effects and interactions were non-significant (*P*s > 0.053), with those involving the training condition all returning *P*-values that were >0.266 (see Fig. 4).

### Summary

In sum, participants in the experimental training condition improved on the face memory task by an average increase of 7.5% over and above those in the control condition, and this was maintained 2 weeks after training terminated. While an improvement in performance was observed between the pre- and post-training assessments for the car memory test, this did not differ between the two training conditions, suggesting it was a task-specific practice effect and supporting the face specificity of training gains. Very little evidence was found for an improvement in face perception, according to both raw accuracy scores and calculation of the face inversion effect. The improvement in face memory scores in the experimental training condition was largest in participants who were weaker at this skill at entry, and also correlated with improvements on upright face perception performance suggesting some shared mechanisms between the two tasks.

## Study 2

Having completed a comprehensive trial of the training programme in adults with prosopagnosia, we assessed the utility of training with a small number of children with face recognition difficulties. Because only a small number of children were available to us and we have already demonstrated the effectiveness of training via controlled trials, we placed all children in the experimental condition. Further, as diagnostic testing for developmental prosopagnosia in children is much less developed than for adults,^4,34^ our inclusion criteria were less rigorous, and we simply aimed to recruit children who reported to us with face recognition difficulties and showed some behavioural evidence of this, as opposed to those who met any formal criteria for prosopagnosia.

### Materials and methods

#### Participants

Parents or guardians of 10 children aged 5–11 years contacted us in the belief that their children have everyday difficulties with face recognition. All were invited to participate in the study over a 7-month period (November 2018–July 2019), and seven completed the training programme during this time period (three children did not engage with training from the outset: aged 6, 8 and 9 years). Three further children were excluded from this report: one child (aged 5 years) completed the training programme but did not wish to complete the post-training assessment tests, and two children (aged 9 and 11 years) performed very well on the pre-assessment tasks and did not fit the basic inclusion criteria for this study (that is, below-average performance on at least one face-processing task). The remaining sample (see Supplementary Table 2 for further background information) of four children (three male) were aged between 5 and 9 years (*M* = 6.8 years, SD = 2.1).

Initial tests of face recognition (pre-training tests or separate assessments) indicated that three of the four children (C01, C02, C03) performed below the fifth percentile for their age on face memory tests; one child (C03) also performed below the second percentile for their age on face matching. The remaining child (C04) performed below the 15th percentile for his age range on both face memory and matching tasks. As such, C03 would meet formal criteria for developmental prosopagnosia^34^; and C01 and C02 may be considered potential cases; whereas C04 presents with milder face recognition difficulties. All children assented to take part in the study, and parental consent was provided according to the Declaration of Helsinki. Ethical approval for the study was granted by the institutional ethics committee.

#### Materials, procedure and statistical analyses

The exact protocols and materials were used as described for the experimental training condition in Study 1. To assess the efficacy of training, we used the same tasks as in our trial with typical children,^25^ all designed for children and known to have adequate reliability.^4^ These tasks essentially parallel the face and object memory and perception tests used in Study 1 but use children’s bicycles rather than cars.

Face memory was assessed with the CFMT-Kids.^44^ The basic procedure of this task is identical to the short version of the CFMT, except that children’s faces are used as stimuli. In line with previous work,^4^ younger children (<7 years) completed a shorter version of the task, with only four target faces (48 trials in total) and older children completed the full version of the task with six target faces (72 trials in total). The Bike Memory Test^4^ follows an identical structure, except all stimuli are bicycle images. To avoid fatigue effects in our youngest participants, the Bike Memory Test was only administered to older children (>7 years) in this study. Bennetts *et al*.^4^ reported Cronbach’s *α* to vary between 0.88 and 0.92 across all the memory tasks. Matching tasks^4^ (Cronbach’s *α* = 0.86–0.87^4^) were used to assess the face and bike perception: children were asked to match a target item (face or bike) to one of three simultaneously presented test items. There were 30 trials for each stimulus type for all participants, regardless of age.

As in Bate *et al*.,^25^ we re-administered the identical tasks at each assessment stage of the study (see below). In line with our approach in Study 1, this is primarily because alternate versions of the tasks have not been developed, but the use of the same tests as Bate *et al*.^25^ facilitate comparisons with the training data collected in typical children (which is used as control data here). Scores on the memory and matching tests were converted to percentage correct. Due to the limited number of participants, analyses were carried out at the individual case level. McNemar tests were used to compare pre- and post-training scores, and pre-training and follow-up data. McNemar tests, which have been used to examine training gains after other face-processing interventions,^17,18,24^ are non-parametric tests that assess the distribution of observations in different categories (similar to *χ*^2^ tests). However, unlike *χ*^2^ tests, McNemar tests can be used to examine paired data^45^—in this case, the paired samples consisted of the performance of each child on pre- and post-training (or follow-up) tests; data for each trial were categorized dichotomously (coded as correct or incorrect). The null hypothesis tested is that the training did not change the distribution of correct and incorrect trials in each test; a significant result (*P* < 0.05) indicates a significant difference between pre-training and post-training (or follow-up) responses.

### Results

#### Engagement and progression

All participants completed the training as instructed (i.e. 30 min per day, on any 10 days within the 14-day period). Two participants (C01 and C03) reached the highest level of training (Level 10). C04 and C02 reached Levels 3 and 4, respectively.

#### Memory

Performance on the face memory tests at each assessment point is shown in Table 2. To determine whether there were any improvements in performance across assessments, McNemar tests examined differences between pre- and post-training scores, and between pre-training and follow-up scores (see Table 2). For comparison, data from typically developing children who previously completed the experimental training^25^ are included.

**Table 2:** Face memory performance pre-training, post-training and at follow-up

Participant	Face memory accuracy (SD)			McNemar test statistics	
	Pre-training	Post-training	Follow-up	Pre versus post	Pre versus follow-up
C01	62.50	75.00	95.83	2.00 (*P* = 0.157)	14.22 (*P* < 0.001)
C02	35.42	41.67	77.08	0.39 (*P* = 0.531)	13.33 (*P* < 0.001)
Younger controls (*N* = 21)^a^	62.10 (20.75)	85.62 (17.69)	88.93 (13.34)		
C03	37.50	48.61	40.28	2.00 (*P* = 0.157)	0.15 (*P* = 0.695)
C04	48.61	79.17	90.28	14.23 (*P* < 0.001)	28.12 (*P* < 0.001)
Older controls (*N* = 19)^a^	67.84 (14.84)	91.52 (7.17)	94.10 (7.87)		

^a^ Control data are from the experimental and control training groups tested by Bate *et al*.^25^

Only one child displayed significant gains in face recognition performance in the post-training assessment (C04). However, three out of the four children with face recognition deficits displayed significant gains in face memory performance in the follow-up assessment (C01, C02, C04). One child showed no significant gains in face recognition performance at either post-training or follow-up assessments (C03). Although somewhat mixed, these results confirm that the majority of children with face recognition deficits displayed significant improvements in face memory scores between the pre-training and follow-up assessments.

To examine the specificity of these effects, we also analysed the bike memory data for the two older children at pre- and post-training assessment (bike memory scores were not collected for younger children, or in the follow-up assessment). C03 showed no improvement in bike memory; he scored 63.89% in both the pre- and post-training assessments. In contrast, C04’s performance improved from 47.2% in the pre-training assessment, to 63.89% in the post-training assessment, *χ*^2^ = 4.80, *P* = 0.028.

#### Perception

Performance on the face and bike matching tests at each assessment point is shown in Table 3. As with face memory, McNemar tests examined differences between pre- and post-training scores, and between pre-training and follow-up scores (see Table 3). Due to the smaller number of trials in the matching task (30 per session), a Yates continuity correction of 0.5 was applied to the McNemar test statistics.

**Table 3:** Face and bike matching performance at pre-training, post-training and follow-up

Participant	Face matching accuracy			McNemar test statistics		Bike matching accuracy^a^		McNemar test statistics
	Pre-training	Post-training	Follow-up	Pre versus post	Pre versus follow-up	Pre-training	Post-training	Pre versus post
C01	86.67	96.67	96.67	3.00 (*P* = 0.083)	3.00 (*P* = 0.083)	70.00	90.00	3.00 (*P* = 0.083)
C02	40.00	66.67	90.00	4.00 (*P* = 0.045)	11.84 (*P* < 0.001)	26.67	73.33	9.80 (*P* = 0.002)
Younger controls (*N* = 21)^b^	86.98 (14.14)	92.06 (13.06)	92.81 (10.61)			74.44 (14.80)	83.17 (14.23)	* *
C03	43.33	43.33	46.67	0 (*P* = 1)	0.06 (*P* = 0.796)	53.33	76.67	2.88 (*P* = 0.089)
C04	63.33	83.33	83.33	6 (*P* = 0.01)	4.5 (*P* = 0.034)	36.67	56.67	3.00 (*P* = 0.083)
Older controls (*N* = 19)^b^	84.03 (7.66)	88.95 (6.48)	90.62 (3.69)			79.47 (9.18)	92.03 (4.29)	* *

^a^ Bike matching data were only collected at pre-training and post-training assessments.

^b^ Control data are from the experimental and control training groups tested by Bate *et al*.^25^ Data show mean (SD) for each group.

One child (C04) showed significant improvements in face matching at post-training; improvements at the follow-up session were marginal. Another child (C02) showed the opposite pattern: marginal improvements in face matching in the post-training session and significant improvements at the follow-up session. Neither C01 nor C03 showed significant improvements in face matching, at either the post-training or follow-up assessments. Only one child (C02) showed significantly increased bike matching performance in the post-training assessment.

#### Gains in everyday life

We did not formally assess face recognition beyond the standardized assessment tasks; however, some parents provided feedback on their child’s face recognition following the training. C01’s parents reported that, following training, ‘he is fine watching films now as before he could only watch cartoons. Also, he started drawing faces with different face features and different expressions’. Before training, C02’s parents reported that she would almost always avoid looking at someone’s face and was very tentative when completing the face tasks; however, in the follow-up assessment sessions, she would concentrate on the faces and make spontaneous comments such as ‘That girl has big eyebrows so that’s easy’ or ‘It’s obviously that one’. In the follow-up assessments, C04 commented that ‘one of the faces was just like an old friend of his’. His parents noted that they have not previously heard him make a confident verbal reference about a face’s identity.

### Summary

In sum, following training, two children (C02 and C04) showed significant gains in both face memory and face matching. It is unclear how face-specific these gains are: both children also showed increased performance in the bike tasks (C02 for bike matching, C04 for bike memory). C01 also showed a significant increase in face memory (but not face matching). Qualitative feedback from the parents of these three participants was in line with these results, reporting higher levels of engagement with faces and better real-world face recognition following training. In contrast, C03 showed no-training gains for either face memory or matching.

## Discussion

This paper aimed to examine the efficacy of a face training programme in adults and children with face recognition difficulties. In Study 1, adults known to have developmental prosopagnosia took part in a randomized placebo-controlled implementation of the programme. Gains were observed in the face but not object memory, with limited evidence for improvement in face perception but not object perception. In Study 2, four children with face recognition difficulties took part in the experimental face training programme. Three of the four children showed gains in face memory, with two also demonstrating gains in face matching, but there was less evidence for face specificity.

Findings from Study 1 largely replicated those from our existing trial with typical children^25^; gains were mostly limited to face memory. The average gain in the adult participants reported here was also remarkably similar to that previously observed in typical children: 7.5 versus 7.6%, suggesting a similar gain from the 2-week training period despite differences in participant age and assessment tasks. Why the gains still did not extend to face perception is unclear given the perceptual rather than mnemonic nature of training, but may result from the assessment tasks used. Indeed, very recent work has evaluated existing face perception tasks that are deemed suitable for use in prosopagnosia, and an emerging consensus is that existing tasks lack both sensitivity and reliability.^46,47^ This has led to the development of some new face perception tasks^46,47^ that unfortunately were not available at the time this study was carried out. Alternatively, the fact that face perception skills were spared in most of the sample (see Supplementary Table 1: only of the 10 individuals in the training condition showed face perception impairments at entry) may also account for the lack of improvements on this measure.

Some insights can be gained into the underpinnings of the gain in face memory. Given the control condition showed no gains whatsoever in face memory between the pre- and post-assessments (*M* = 55.20 versus *M* = 55.88%), it seems unlikely that more generalized non-perceptual processes were strengthened, as both conditions employed the same overall task (e.g. face–name association of abstract stimuli). Rather, the experimental condition aimed to pull attention towards discrete differences in expression, feature size or feature spacing—manipulations that have both been found to bring about modest gains in the previous face training studies.^19,21,22^ The finding that CFMT+ performance particularly improved on the noise trials of the task tentatively suggests that the training may have acted on configural processing mechanisms, although this was not supported by the CFPT data. By including both upright and inverted versions of the CFPT, we were able to examine improvements in holistic and configural processing for face perception. Sensitivity to feature spacing information is particularly disrupted by inversion,^42^ so we expected that training individuals to attend to spacing cues may increase their inversion effect. However, our results did not support this prediction—there was no evidence that the training increased the inversion effect for faces. It is possible that participants in the experimental training condition focused more on features (as these were also manipulated in the training stimuli); alternatively, it may be that changes in face-processing strategies following training are more subtle, and detecting them would require higher numbers of participants or a longer duration of training. For example, DeGutis *et al*.^19^ detected changes in the face inversion effect following 15 uninterrupted days of training (compared with 10 days within a fortnight in the current study). As noted above, it is also possible that perceptual gains (both overall and in relation to the inversion effect) were minimal in this study because many participants did not show severe deficits in face perception.

The results of Study 2 provide preliminary support for the idea that *Guess Who*-style training may also improve face recognition in children with face recognition impairments: three out of the four children who completed the training showed a significant improvement in face perception and/or memory at follow-up testing. Unlike Study 1, post-training improvements (either at the post-training or follow-up sessions) were observed in both the memory tasks (three participants) and the perception tasks (two participants). This may reflect the different analytical approaches in Studies 1 and 2, or the fact that face perception deficits are more prevalent in children than adults with developmental prosopagnosia.^44^

The face training gains varied substantially between participants (from <4 to 50%), suggesting substantial heterogeneity in this group. It is unclear which factors predict training gains in this population, but an examination of pre-training scores (on both face and object processing tasks) suggests that they are not strongly associated with training gains.

Due to the relatively small number of children with face recognition deficits, and the difficulty of engaging these children in training programmes, we did not have a control group for Study 2. Consequently, it is not possible to dissociate the gains in performance associated with the experimental training from that associated with practice or control training effects. Previous findings with children of a similar age suggest that, in typical children, the training improves face recognition over and above the effects of the practice.^25^ However, unlike our previous work, which found minimal gains in object recognition,^25^ several children in this sample showed significantly improved bike memory or matching following training. This could indicate that the improvements in the face tasks are simply practiced effects. We did not select the children on the basis of their object recognition abilities, and it is notable that the children who showed improvement in the bike tasks performed particularly poorly in the pre-training object assessments. As such, it may be possible that those children showed more general perceptual deficits. It is possible that the more generalized gains arose because children who perform poorly at baseline simply have more room to improve—in other words, practice effects (for both faces and objects) may be more pronounced and easier to detect in these children. Alternatively, it is possible that the training affected more general perceptual processes, and results in domain-general gains in these children. Importantly, though, this also suggests that the training programme may be effective for children with broader perceptual deficits.

Critically, the face-specific effects of training in Study 1 indicate at last some plasticity in the face recognition systems of adults with developmental prosopagnosia. While such a firm conclusion cannot be drawn from the smaller sample size of children, these findings provide promising evidence that this face training programme can bring about improvements in face memory after only 2 weeks of participation, and those gains are maintained for at least 2 weeks after training terminates. Clearly though, larger and more long-lasting benefits in both adults and children with face recognition difficulties will be garnered from longer-term training (the study performed by DeGutis *et al*.^19^ required more engagement over a 3-week period, whereas the training period was 11 weeks in both Corrow *et al*.^22^ and Davies-Thompson *et al*.).^21^ The *Guess Who* format provided an engaging means of face training for children without requiring screen time, but the training could be repackaged into a more adult-appropriate online format that does not require a human opponent. Further, it is notable that both adult and child participants reached the highest possible level within the 2-week period, and increased difficulty or alternative playing cards would be required for the sustainability of longer-term training.

While future work should address these developments and continue to test further samples of adults and children with prosopagnosia as they become available, the current study has provided promising evidence for further implementations of this face training programme, while presenting important evidence supporting plasticity and domain-specificity through to mid-adulthood in cases of developmental prosopagnosia.

## Supplementary Material

fcac068_Supplementary_Data

## Abbreviations

CFMT =: Cambridge Face Memory Test
CFPT =: Cambridge Face Perception Test
