# Supplementary material for: Face recognition improvements in adults and children with face recognition difficulties
Source: Brain Commun. 2022 Mar 22;4(2):fcac068. doi: 10.1093/braincomms/fcac068 (PMC8977649; doi:10.1093/braincomms/fcac068)
Supplement: fcac068_Supplementary_Data [file fcac068_supplementary_data.docx]

**Supplementary Table 1: Pre-study diagnostic scores for the adults with developmental prosopagnosia in Study 1.** All participants had contacted our laboratory prior to participating in the study in the belief that they experience severe difficulties with face recognition. In line with dominant protocols in the literature,^7,34^ our inclusion criteria were set at 1.7 from an age-matched control mean on at least two of three screening tests: the CFMT, the CFPT, and a famous face recognition test used in our previous work.^7^

| **Participant** | **Age** | **Gender** | **CFMT (%)** | **CFPT (upright %)** | **Famous Faces (%)** |
| --- | --- | --- | --- | --- | --- |
| Experimental: |  |  |  |  |  |
| E01 | 25 | F | 43.06* | 66.67 | 31.48* |
| E02 | 39 | M | 59.72* | 69.44 | 28.33* |
| E03 | 51 | M | 58.33* | 72.22 | 75.00* |
| E04 | 55 | M | 43.06* | 58.33 | 30.23* |
| E05 | 30 | M | 38.89* | 52.78* | 45.00* |
| E06 | 39 | F | 58.33* | 51.39* | 33.90* |
| E07 | 37 | F | 52.78* | 66.67 | 54.90* |
| E08 | 27 | F | 45.83* | 55.56* | 33.33* |
| E09 | 57 | M | 45.83* | 62.50 | 31.03* |
| E10 | 52 | F | 48.61* | 51.39* | 25.00* |
| Control: |  |  |  |  |  |
| C01 | 54 | F | 59.72* | 62.50 | 57.14* |
| C02 | 58 | F | 48.61* | 50.00* | 81.40 |
| C03 | 56 | F | 48.61* | 80.56 | 71.43* |
| C04 | 43 | M | 58.33* | 70.83 | 62.50* |
| C05 | 56 | F | 54.17* | 58.33 | 63.79* |
| C06 | 54 | F | 45.83* | 62.50 | 55.93* |
| C07 | 38 | F | 45.83* | 55.56* | 47.83* |
| C08 | 31 | F | 61.11* | 65.28 | 67.27* |
| C09 | 33 | F | 56.94* | 73.61 | 55.93* |
| a | 33 | F | 55.56* | 63.89 | 47.37* |

*score exceeds 1.7 SDs from the relevant age-matched control mean

**Supplementary Table 2: Individual case summaries of children with face recognition difficulties in Study 2.** Face recognition performance was only assessed as part of the training study unless reported otherwise. Performance at pre-assessment confirmed inclusion for this study (see manuscript). Percentiles are based on age-matched control performance as reported in ^4^

| **ID** | **Gender** | **Age** | **Baseline face memory** | **Baseline face matching** | **Notes** |
| --- | --- | --- | --- | --- | --- |
| C01 | M | 5 | 27.08%  (2^nd^-5^th^ percentile)* | 86.67%  (85^th^-95^th^ percentile) | Diagnosed with Developmental Language Disorder, characterised by severe receptive and expressive language delays. Also exhibits some social communication issues, and was awaiting an assessment for ASD at the time of training. Demonstrates above-average performance at many non-verbal standardised tasks (e.g., visual reasoning-based tasks), with the exception of visual-spatial short-term memory. Completed an assessment for prosopagnosia with one of the authors prior to commencing training. |
| C02 | F | 5 | 35.42%  (2^nd^-5^th^ percentile) | 40.00%  (10^th^-15^th^ percentile) | Assessed for ASD by family paediatrician but did not meet diagnostic criteria. Parents report no visual difficulties except face recognition, and no history of neurological injuries or illness. |
| C03 | M | 8 | 37.50%  (<2^nd^ percentile) | 43.33%  (<2^nd^ percentile) | Completed an assessment for ASD with local psychologists but did not meet diagnostic criteria. Parents did not report any other neuropsychological disorders, developmental disorders, or problems with vision. Uses hairstyle and voice to distinguish between people; parents noted that he often misidentifies people with similar hairstyles. |
| C04 | M | 9 | 48.61%  (10^th^-15^th^ percentile) | 63.33%  (10^th^-15^th^ percentile) | Received a diagnosis of ASD in the year before training commenced. Parents reported mild problems with vision (longsightedness), although not sufficient to disrupt everyday functioning. |

* Data from prior assessment with authors.
